# Wound dehiscence: is still a problem in the 21th century: a retrospective study

**DOI:** 10.1186/1749-7922-4-12

**Published:** 2009-04-03

**Authors:** John Spiliotis, Konstantinos Tsiveriotis, Anastasios D Datsis, Archodoula Vaxevanidou, Georgios Zacharis, Konstantinos Giafis, Spyros Kekelos, Athanasios Rogdakis

**Affiliations:** 1 Department of Surgrery, Messologi General Hospital, Messologi, Greece; 2 Department of Anesthesiology, Messologi General Hospital, Messologi, Greece; 3 Intensive Care Unit, Arta General Hospital, Arta, Greece

## Abstract

**Background:**

The aim of this study was to evaluate the risk factors of wound dehiscence and determine which of them can be reverted.

**Methods:**

We retrospectively analyzed 3500 laparotomies. Age over 75 years, diagnosis of cancer, chronic obstructive pulmonary disease, malnutrition, sepsis, obesity, anemia, diabetes, use of steroids, tobacco use and previous administration of chemotherapy or radiotherapy were identified as risk factors

**Results:**

Fifteen of these patients developed wound dehiscence. Emergency laparotomy was performed in 9 of these patients. Patients who had more than 7 risk factors died.

**Conclusion:**

It is important for the surgeon to know that wound healing demands oxygen consumption, normoglycemia and absence of toxic or septic factors, which reduces collagen synthesis and oxidative killing mechanisms of neutrophils. Also the type of abdominal closure may plays an important role. The tension free closure is recommended and a continuous closure is preferable. Preoperative assessment so as to identify and remove, if possible, these risk factors is essential, in order to minimize the incidence of wound dehiscence, which has a high death rate.

## Background

Surgical wound dehiscence after laparotomy remains a serious complication. It presents a mechanical failure of wound healing of surgical incisions. Surgical incisions stimulate the healing process which in reality is a complex and continous process with four different stages: Hemostasis, inflammation, proliferation, and maturation [1].

During hemostasis, platelets aggregate, degranulate and activate blood clotting. The clot is degrading, the capillaries dilates and fluids flow to the wound site, activating the complement cascade.

Macrophages, lysis of cells and neutrophills are a source of cytokines and growth factors that are essential for normal wound healing [1,2].

The proliferation phase which is the phase of granulation tissue forms in, the wound space begins in the 3 postoperative day and lasts for several weeks.

The most important factor in this phase are fibroblasts which move to the wound and are responsible for the collagen synthesis [3,4]. The maturation phase begins in the 7 postoperative day and lasts for 1 year or more, continued collagen deposition and remodeling contribute to the increased tensile strength of wounds.

Post laparotomy wound dehiscence occurs in 0,25% to 3% of laparotomy patients and immediate operation is required which has a death rate of 20% [2,5,6].

Conditions associated with increased risk of wound dehiscence are anemia, hypoalbuminemia, malnutrition, malignancy, jaundice, obesity and diabetes, male gender, elderly patients and specific surgical procedures as colon surgery or emergency laparotomy which are associated with wound disruption [7,8].

The aim of this study is to evaluate retrospectively the risk factoers of wound dehiscence and to determine which of them can be revert.

## Methods

Between 2001 and 2007, 3500 abdominal laparotomies were performed in the Department of surgery of Mesologgi General Hospital and urban community teaching hospital of 150 beds.

Fifteen patients were reported with complete wound dehiscence.

The medical reports of all patients were reviewed and local, systemic, operative factors were compared (Factor analysis)

1. Age > 70 years are described as risk factor

2. Malignancy, the presence of malignancy during the operation is estimated as a risk factor.

3. COPD, the medical history of COPD or the PO2 < 60 and PCO2 < 30 also estimate as a risk factor.

4. Malnutrition, the total serum albumin level less than 3,0 mg/dl and the decrease of body weight more than 10% in the last 10 months are estimated as risk factors

5. The presence of Sepsis

6. Obesity, BMI > 35

7. Radiotherapy or chemotherapy treatments before operation are described as risk factors

8. Anemia, Hb < 10 mg/dl is described as risk factor

9. Diabetes is described as risk factor

10. Steroid treatment in the last 12 months are estimated as risk factor.

11. Operative factors such as type of operation, suture materials and postoperative morbidity were compared.

## Results

Fifteen of 3500 patients developed wound dehiscence (0,43%)

The primary diagnoses and initial operative procedures that concluded to wound dehiscence are listed in table 1.

**Table 1:** Diagnosis and operative procedure of the patients with wound dehiscence.

Diagnosis n	Operative procedure n
Ulcer perforation = 3	Simple closure = 3

Acute cholecystitis = 2	Cholecystectomy = 2

Colon cancer = 5	Right colectomy = 3 Abdominoperineal resection = 2

Intestinal obstruction = 2	Small intestine resection = 2

Abdominal abscess = 2	Small intestine resection = 2 Appendectomy = 1

Liver Hydatide cyst = 1	Cystectomy = 1

In the 9 of these15 patients (60%) emergency laparotomy was performed.

The mean age was 69,5 years (ranging fro 55 to 81) and 9 of them (60%) are male.

The risk factors and the final outcome are listed in table 2.

**Table 2:** Patients risk factors concerning the medical history

n	Sex	Age	Cancer	COPD	Malnutrition	Sepsis	Obesity	Radio/Chemo	Anemia	Diabetes	Steroid	Total risk factor	Outcome
1	M	71	-	+	-	+	+	-	-	+	-	4/10	Surv.

2	F	74	+	+	+	-	-	+	+	-	+	7/10	Surv.

3	M	81	-	+	+	+	-	-	+	+	+	7/10	Died

4	M	74	+	-	-	+	-	+	-	-	-	4/10	Surv.

5	F	67	+	+	-	-	-	-	-	+	-	3/10	Surv.

6	M	55	-	-	-	+	+	-	-	+	-	3/10	Surv.

7	F	76	+	+	-	+	+	+	-	+	-	7/10	Died

8	M	56	-	+	-	+	+	-	-	-	-	3/10	Surv.

9	F	73	+	-	+	-	-	+	+	-	-	5/10	Surv.

10	M	72	-	+	-	-	-	+	+	-	-	4/10	Surv.

11	M	78	+	+	+	+	-	+	+	-	+	8/10	Died

12	M	71	-	-	-	-	-	-	-	+	-	2/10	Surv.

13	M	64	-	+	-	-	+	-	-	-	-	2/10	Surv.

14	F	68	+	+	-	-	-	-	+	-	-	3/10	Surv.

15	F	74	+	-	+	+	-	-	-	-	-	4/10	Surv.

Elderly patients and history of COPD are present in the 67% of cases, cancer and sepsis in the 53,3% of cases.

The presence of anemia, diabetes mellitus and the history of received chemotherapy or radiotherapy are 40% in iur patients.

Malnutrition and obesity are present in one third of our patients.

Only 20% of patients did receive treatment with steroids in the last 12 months.

Concerning the surgical history and the postoperative morbidity, the results are listed in table 3.

**Table 3:** Patients surgical characteristics and postoperative outcome

n	Incision	Wound closure	Drain	Postoperative Complication	Wound dehiscence observed Postoperative day
1	Kocher	Separate closure	No	No	6

2	Midline	Separate closure	Yes	No	9

3	Midline	Separate closure	Yes	Pneumonia	14

4	Midline	Separate closure	Yes	No	9

5	Midline	Separate closure	Yes	No	7

6	Midline	Separate closure	Yes	No	8

7	Midline	Continuous closure	No	Fistula	7

8	Kocher	Separate closure	No	Intraabdominal Sepsis, Abscess	9

9	Mercedes	Separate closure	Yes	No	16

10	Kocher	Separate closure	No	No	14

11	Midline	Continuous closure	Yes	No	7

12	Midline	Separate closure	Yes	Catheter Sepsis	6

13	Midline	Continuous closure	Yes	No	9

14	Midline	Continuous closure	Yes	Catheter Sepsis	9

15	Midline	Continuous closure	Yes	Pneumonia	8

Wound dehiscence was more often observed on the 9,2 postoperative day (ranging from the 6^th ^to 15^th^).

Three patients (20%) developed wound dehiscence after their initial discharge and were readmitted to our hospital.

Concerning the type of incision or the abdominal closure, only the presence of interrupted suturing of linea alba (10/14) patients plays a role in the wound dehiscence. This factor factor is a hypoestimated parameter in he past as a possible risk factor.

All patients are reoperated after the wound dehiscence diagnosis and three of them (20%) died due to postoperative complication of reoperation. In one of them recurrence of wound dehiscence was observed. Regarding the preoperative risk factors, three from four (75%) patients with 7 or more risk factors did die. The abdominal closure was performed using mesh in 4 cases, a flap in 2 cases and a continuous suturing in 9 cases. Retention suture were used in 2 cases.

## Discussion

Wound dehiscence is a mechanical failure of wound healing, remains a problem and it can be affected by multiple factors.

Pre-operative conditions especially in elective operations should be recommended to reduce or eliminate the risk. No tobacco use, no steroid use prior to surgery, carefully controls of the patients comorbidity as anemia, malnutrition, obesity and cardiovascular or lung diseases.

During the surgical procedures, measure to reduce the risk of infections and hypoxia in the tissue are the to most importants factors for the postoperative wound healing process.

The type of abdominal closure may plays an important role. The tension free closure is recommended and a continuous closure is preferable.

Our study in accordance with other reports [6,8-10] demonstrates a significantly higher incidence of postoperative wound dehiscence in emergency than in elective surgery.

It is important for the surgeon to knows that wound healing demands oxygen consumption, normoglycemia and absence of toxic or septic factors, which reduces collagen synthesis and oxidative killing mechanisms of neutrophils [11,12]

Wounds heal by primary, secondary or tertiary intention, wounds that are approximated heal by primary intention mainly by deposition of connective tissue.

The important observation is that wounds which are left to heal by secondary intention are dehiscent frequently because these heals more slowly due to amount of connective tissue

That is necessary to fill the wound [13].

Management of dehisced wounds may include immediate re-operation if bowel is protruding from the wound. Mortality rates associated with dehiscence have been reported between 14–50% [3]. In our study mortality rate is 20%.

On the other hand the best case scenario is a discharging wound which leads to the appearance of an incisional hernia.

## Conclusion

In conclusion in re-operation certain strategies, such as using a vacuum assisted closure in patient with compromised healing (6) or using tension free mesh techniques in order to reduce the tension of the abdominal wall.

## Competing interests

The authors declare that they have no competing interests.

## Authors' contributions

SJ, TK, DA, VA, ZG, GK, KS and RA have all made substantial contributions to conception and design, acquisition of data or analysis and interpretation of data.
